# Organic buffers act as reductants of abiotic and biogenic manganese oxides

**DOI:** 10.1038/s41598-023-32691-5

**Published:** 2023-04-20

**Authors:** Debra M. Hausladen, Jasquelin Peña

**Affiliations:** 1 Institute of Earth Surface Dynamics, University of Lausanne, 1015 Lausanne, Switzerland; 2 Department of Civil and Building Engineering, Université de Sherbrooke, Sherbrooke, QC J1K 2R1 Canada; 3 Department of Civil and Environmental Engineering, University of California, Davis, CA 95616 USA

**Keywords:** Biogeochemistry, Environmental sciences, Geochemistry, Environmental chemistry, Geochemistry

## Abstract

Proton activity is the master variable in many biogeochemical reactions. To control pH, laboratory studies involving redox-sensitive minerals like manganese (Mn) oxides frequently use organic buffers (typically Good’s buffers); however, two Good’s buffers, HEPES and MES, have been shown to reduce Mn(IV) to Mn(III). Because Mn(III) strongly controls mineral reactivity, avoiding experimental artefacts that increase Mn(III) content is critical to avoid confounding results. Here, we quantified the extent of Mn reduction upon reaction between Mn oxides and several Good’s buffers (MES, pK_a_ = 6.10; PIPES, pK_a_ = 6.76; MOPS, pK_a_ = 7.28; HEPES, pK_a_ = 7.48) and TRIS (pK_a_ = 8.1) buffer. For δ-MnO_2_, Mn reduction was rapid, with up to 35% solid-phase Mn(III) generated within 1 h of reaction with Good’s buffers; aqueous Mn was minimal in all Good’s buffers experiments except those where pH was one unit below the buffer pK_a_ and the reaction proceeded for 24 h. Additionally, the extent of Mn reduction after 24 h increased in the order MES < MOPS < PIPES < HEPES << TRIS. Of the variables tested, the initial Mn(II,III) content had the greatest effect on susceptibility to reduction, such that Mn reduction scaled inversely with the initial average oxidation number (AMON) of the oxide. For biogenic Mn oxides, which consist of a mixture of Mn oxides, bacterial cells and extracelluar polymeric substances, the extent of Mn reduction was lower than predicted from experiments using abiotic analogs and may result from biotic re-oxidation of reduced Mn or a difference in the reducibility of abiotic versus biogenic oxides. The results from this study show that organic buffers, including morpholinic and piperazinic Good’s buffers and TRIS, should be avoided for pH control in Mn oxide systems due to their ability to transfer electrons to Mn, which modifies the composition and reactivity of these redox-active minerals.

## Introduction

Proton activity is the master variable in most biogeochemical processes and reactions that occur at water-particle interfaces. For layer-type Mn oxides (MnO_x_), which are ubiquitous in a range of terrestrial and aquatic environments^1–3^, the kinetics and extent of contaminant oxidation and sorption, as well as mineral properties such as interlayer cation content, crystallite size, aggregation and ability to undergo phase transformation, are strongly dependent on suspension pH^4–7^. Therefore, studying interfacial processes involving MnO_x_ requires pH control, which is usually achieved with inorganic (e.g., phosphate^8^, carbonate^9–11^, borate^12,13^) or organic buffers (most popularly Good’s buffers)^14,15^. Although inorganic buffers are generally resistant to oxidation, they may influence mineral reactivity through the formation of surface complexes or removal of free metal ions from solution through aqueous complexation or precipitation reactions.

Good’s buffers are *N*-substituted aminosulfonic acids that were developed as alternatives to pH buffers such as phosphate and TRIS (tris(hydroxymethyl)aminomethane), which either have poor buffering capacity under physiological pH conditions and/or interact with metals through complexation, precipitation or oxidation reactions^14^. MES, (2-(*N*-morpholino)ethanesulfonic acid) along with MOPS (3-(*N*-morpholino)propanesulfonic acid) and PIPES (piperazine-*N*,*N*′-bis(2-ethanesulfonic acid)), are the three of twenty well-known Good’s buffers that were proposed to not complex metal ions^16^; other Good’s buffers are known to interact with hydrated metal ions forming bidentate chelate rings using one alcoholic oxygen and the nearest amine group^17^. Piperazine-ring containing buffers like HEPES (4-(2-hydroxyethyl)-1-piperazineethanesulfonic acid) form radical species and thus are reactive toward redox-sensitive metals^18,19^. HEPES, with a pK_a2_ of 7.48, is one of the most commonly used Good’s buffers, largely due to its ability to buffer pH over a range relevant to natural systems^17^. HEPES is also used in microbial growth media in the study of Mn biomineralization and in the production of biogenic Mn oxides for use in biogeochemical studies^3,20,21^. While the biochemistry literature warned against the use of Good’s buffers in the study of redox-sensitive processes more than two decades ago^17,18^, the environmental science community has been slow to adopt these findings^16,22–35^. Myriad studies involving iron and manganese oxides have employed high Good’s buffer concentrations (10–30 mM)^36–41^, although some recent studies have acknowledged buffer-induced reduction of metals^42–47^.

Good’s buffers continue to be integral in laboratory experiments despite evidence that they complex metals and promote redox transformations of metal oxides^16,18,41–43^. Most notably, characterization of redox-sensitive biogenic Mn oxides comes primarily from those synthesized in HEPES buffer^20,21,48–57^. Recent studies investigating metal sorption capacity of δ-MnO_2_ inadvertently found that the presence of HEPES reduced Mn(IV) to Mn(III), the accumulation of which lowers the sorption and oxidation capacity of Mn oxides. Simanova et al.﻿^58^ showed that as the Mn(III)-content in δ-MnO_2_ increased upon equilibration with HEPES buffer, nickel sorption capacity decreased; similar trends have been observed for other metals such as cobalt^6,46^. Elzinga and Kustka^44^ also found that HEPES buffer reduced lattice Mn(IV) in δ-MnO_2_, and Hinkle et al.^47^ observed that the average manganese oxidation number (AMON) of c-disordered H^+^ birnessite (a layer-type Mn oxide with hexagonal sheet symmetry and a 10–15% Mn(III) content) decreased in the presence of MES buffer. Furthermore, Good’s buffers have been shown to promote a change in the sheet symmetry of Mn oxides by supplying protons to the oxide surface through the dissociation of the protonated buffer^59^, which favors the displacement of Mn(III) from layer to interlayer crystallographic positions.

Despite an increasing awareness of HEPES interference in redox processes, no study has compared the extent to which organic buffers used across a range of pH values promote reduction of redox-active minerals. Organic buffers from the Good’s classification and amine-based buffers like TRIS contain redox-active moieties in the form of hydroxyl and amine functional groups that may readily react with Mn oxides given their high reduction potentials^1^. Without a clear baseline of how different buffers can impact electron transfer across treatments, mechanistic studies of mineral redox reactivity will be limited^24,32,38,39,43^. Recent studies indicate that average Mn oxidation number in manganese oxides depends on a range of factors, yet the extent to which reduction by organic buffers varies with mineral structure, Mn(III)-content, buffer to total manganese in the solid phase (buffer: Mn_TOT_) ratio, pH, and/or presence of microbial biomass is not known. This is the first study to explore these factors. In addition, we synthesize biogenic Mn oxides without any reductive interference of organic buffers and assess their sensitivity to reduction by HEPES buffer, which has been universally employed in the synthesis of biogenic Mn oxides.

**Figure 1:**
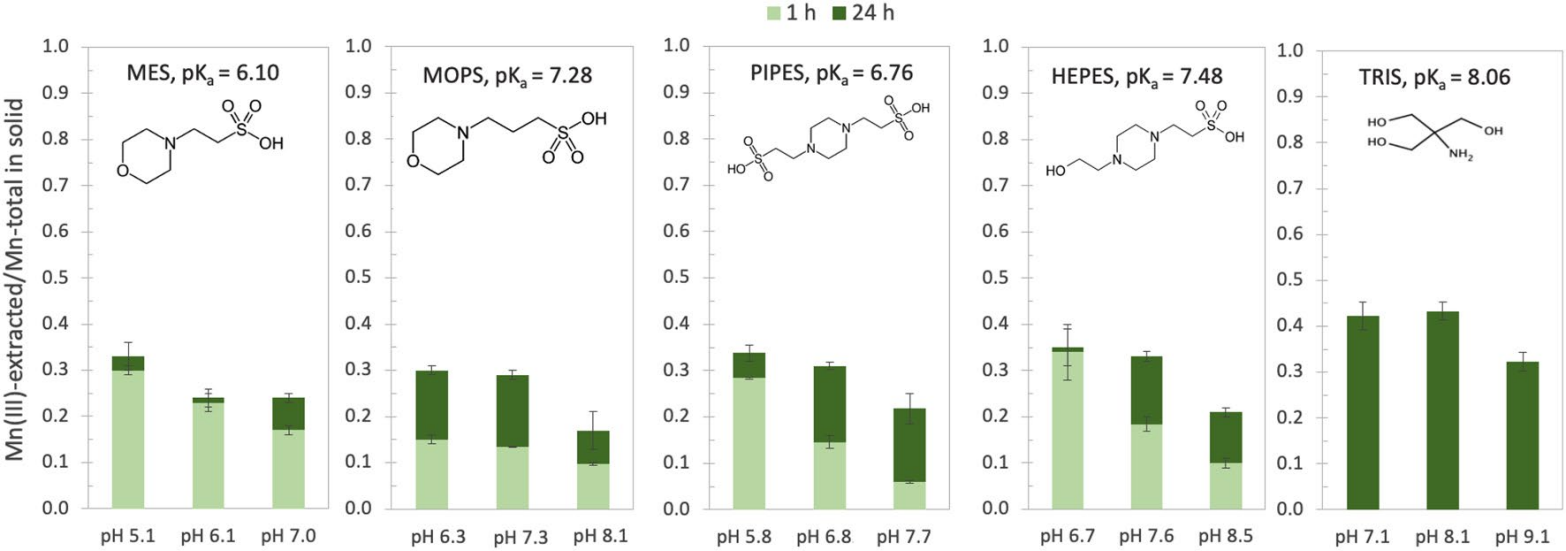
Generation of Mn(III) in δ-MnO_2_ (t = 0: AMON = 4.0) after reaction with 10 mM MES-, MOPS-, PIPES-, HEPES-, and TRIS-buffer as a function of pH (± 1 pH unit from pK_a_) after 1 h (light green) and 24 h (dark green). Pyrophosphate extractions were used to quantify Mn(III) generated upon reaction with all buffers except TRIS. The Mn(III) content of TRIS-reacted δ-MnO_2_ was estimated from AMON values; no 1 h data are available for experiments with TRIS buffer. Error bars represent standard deviation of triplicates for all except TRIS where *n* = 2. The pK_a_ values indicated are for 25 °C.

In this study, we determined the extent of Mn reduction by organic buffers from the morpholinic (i.e., MES and MOPS), piperazinic (i.e., HEPES and PIPES), and TRIS families (Fig. 1, Table S1), which provide pH control over environmentally relevant values. We compared the reducibility of a range of solids, including chemically-synthesized and biogenic Mn oxides as well as oxides with varying initial Mn(III) content. Our results show that none of these organic buffers are suitable for use in the study of redox-sensitive minerals or to investigate redox processes in water–mineral systems.

## Results and discussion

### Reduction of δ-MnO_2_ by common organic buffers

The δ-MnO_2_ used in this study had an initial average manganese oxidation number (AMON) of 4.01 ± 0.01, which is consistent with the Mn(III) pyrophosphate-extraction values and indicates minimal Mn(III) (Table 1). Reaction of δ-MnO_2_ with all buffers at pH values equal to the pK_a_, pK_a_ + 1, and pK_a_-1 led to extensive reduction of Mn(IV) in δ-MnO_2_ (Fig. 1, Supplementary Table S2). The solid-phase Mn(III) content generally increased with decreasing pH relative to the pK_a_ for a given buffer. This trend was prominent in the 1 and 24 h data for MES, MOPS, PIPES, and HEPES experiments (Fig. 1) and is consistent with increasing reduction potential at decreasing pH values^1^. At lower pH values, where surface interactions between the protonated buffers and negatively charged mineral surface are more favourable, the reaction may be kinetically enhanced, allowing those treatments to reach steady state more quickly^60–62^. Given that Mn(II) oxidation by dissolved oxygen may be relevant at the timescale of the reaction for pH values > 8.5^63^, surface catalysed oxidation of Mn^2+^ by oxygen may contribute to the decreased Mn reduction observed at the highest pH treatments (pK_a_ + 1) for HEPES- and TRIS-reacted δ-MnO_2_.

**Table 1:** Properties of synthetic Mn oxides used in this study.

	AMON	Mn(III) (%)	Mn(II) (%)	Mn(IV) (%)	SSA (m^2^ g^−1^)
δ-MnO_2_	4.01 ± 0.01	3.0 ± 1.4	–	97.0 ± 1.4	202
δ-MnO_2_*		13.4 ± 1.4	–	86.6 ± 1.6	–
δ-MnO_2_**		15.2 ± 0.3	–	84.8 ± 0.5	–
c-dis Bi	3.76 ± 0.01	15.6 ± 0.07	4.2 ± 1.5	80.25 ± 0.5	369

A 3-step titration was used to determine AMON values, while pyrophosphate extractions were used to quantify solid-phase Mn(III). The Mn(IV) and Mn(II) contents of c-disordered birnessite (c-dis Bi) were calculated from AMON and solid-phase Mn(III) content; for δ-MnO_2_, the Mn(II) content was below detection as the AMON is equal to 4.01. For δ-MnO_2_* and δ-MnO_2_**, the Mn(IV) content was calculated assuming no solid-phase Mn(II) as observed for δ-MnO_2_. Uncertainty in the calculated values was determined as the weighted sum of the variance, while the error for measured values is given as the standard deviation among replicates.

The reduction of δ-MnO_2_ by HEPES, PIPES, MOPS, and MES generated mostly solid-phase Mn(III), with HEPES buffer reducing up to 35% of the initial Mn to solid-phase Mn(III) at pH 6.7 after 24 h and reduction by the other buffers following closely behind (Fig. 1). For HEPES we found good agreement between the pyrophosphate-extractable Mn(III) and the AMON value, therefore we assume minimal sorbed Mn(II) (Supplementary Table S2). Measurable amounts of Mn_aq_ were only observed in Good’s buffer treatments where pH < 7 (Supplementary Table S2). Most cases generated less than 4 µM aqueous Mn after one hour of reaction; however, after 24 h, we detected 20–50 μM aqueous Mn for reactions run at pH values equal to 1 unit below the pK_a_ for all buffers except MOPS, which generated minimal aqueous Mn (< 1% or 4.1 µM; ~ 410 µM Mn_tot_) under all conditions tested. For MES, PIPES, and HEPES we measured 23, 49 and 21 μM aqueous Mn, respectively (these reactions were run using about 503, 1030, 853 µM Mn_tot_; see Supplementary Table S2). As aqueous Mn(III) is unstable unless in the presence of high-affinity complexing agents with multiple functional moieties (e.g., pyrophosphate, desferrioxamine B, EDTA)^64^, we assume that aqueous Mn represents Mn(II) although the formation of any aqueous Mn(III)-organic species was not measured and published stability constants for Mn(III)-buffer complexes are not available. The reductive dissolution of Mn and its accumulation as Mn(II)_aq_ may occur when the amount of solid-phase Mn(III) is high enough to favor disproportionation to Mn(II) and Mn(IV) and when pH is low enough to limit Mn(II) adsorption or surface-catalyzed oxidation^44,65,66^.

Of the five buffers tested, TRIS led to the greatest reduction of δ-MnO_2_, including the accumulation of aqueous Mn in solution (Fig. 1, Supplementary Table S2). While aqueous Mn concentrations were low (< 2% of the initial Mn_TOT_) for all pH values tested within the first hour of reaction, up to 15 mol% of the initial Mn_TOT_ or 185 μM aqueous Mn accumulated at pH 7.07 after 24 h (Supplementary Tables S2, S4). At pH 8.07 and 9.07, Mn_aq_ did not increase measurably between 1 and 24 h, which is consistent with favorable sorption of Mn(II, III) and surface-catalyzed oxidation of Mn(II) at these pH values^65,67,68^. For the solid phase, we measured an AMON value of 3.60 at pH 7.1, which would indicate about 40% Mn(III), under the assumption of minimal Mn(II) sorption. In separate experiments, we measured about 776 μM PP-extractable Mn(III) from the solid phase (~ 72%). This large discrepancy between AMON and PP-extractable Mn(III) may arise from continued reaction between adsorbed TRIS and Mn(III,IV) during the PP extraction reaction, especially as unintended reductive dissolution of the solid phase was observed (i.e., aqueous Mn measured by ICP-OES was greater than Mn(III)-PP determined by UV–Vis spectrophotometry, (Supplementary Tables S2, S4)). Given the difficulty in quantifying the accumulation of Mn(II, III) in these experiments and the propensity of TRIS to form complexes with Mn, the rest of the experiments focused on Mn interactions with the four chosen Good’s buffers.

**Figure 2:**
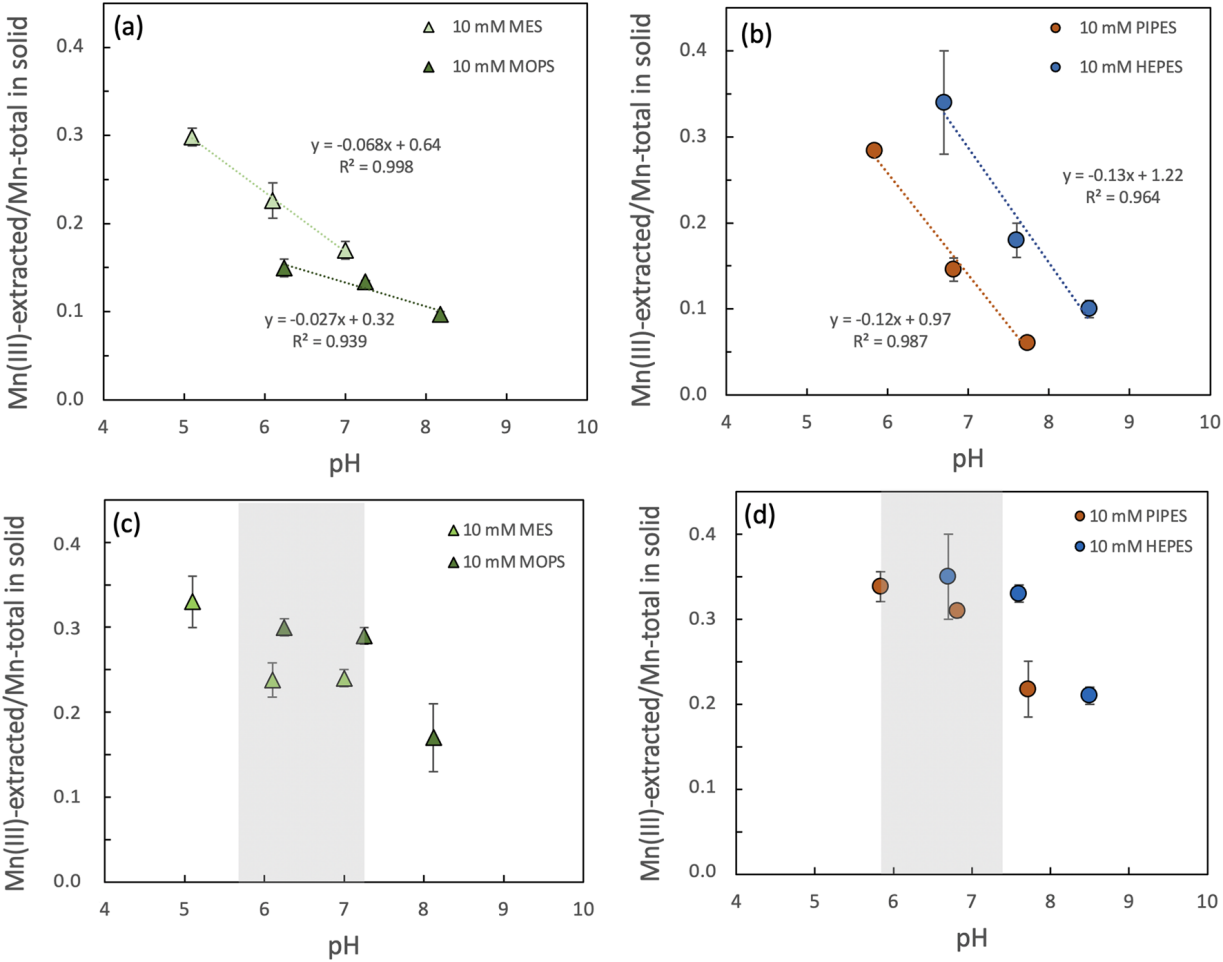
Pyrophosphate-extracted Mn(III)-content of δ-MnO_2_ reacted with buffers containing a morpholine (**a, c**) versus piperazine ring (**b, d**) as a function of pH after 1 h (**a, b**) and 24 h (**c, d**). Linear regressions show similar slopes for similar buffer structure after 1 h. At 24 h, grey bars denote the circumneutral pH region where Mn reduction is less responsive to pH. Error bars represent standard deviation of triplicates, when not visible error bars are smaller than the marker size.

In Fig. 2, we compare the effect of pH on Mn(III) generation for the morpholinic- vs. piperazinic-ring containing buffers. Within the first hour of reaction, solid-phase Mn reduction was less impacted by pH upon reaction with buffers contanaining a morpholine ring (MES and MOPS; Fig. 2a) than upon reaction with buffers containing a piperazine ring (PIPES and HEPES; Fig. 2b), as indicated by the lower slopes at 1 h. After 24 h of reaction, the effect of pH rather than the buffer structure appears to dominate trends in Mn reduction. The Mn(III) content was approximately constant in the circumneutral pH range but increased and decreased under acidic and alkaline conditions, respectively (Fig. 2c,d). The trends observed for MES and MOPS *vs*. PIPES and HEPES may be explained by the presence of oxygen within the morpholine ring, which may produce an electron withdrawing effect on the ring-bound N and reduce its susceptibility to oxidation^69^.

Based on the presence of oxygen in the morpholine ring and the absence of the hydroxyl branch of HEPES^17,69–71^, we expected that MOPS and MES buffers would be less reactive than HEPES. For example, in an investigation of the oxidative capacity of Mn oxides, Pan et al. selected MOPS as a less reactive alternative to HEPES^32^; however, control experiments simply used the absence of MnO_2_ dissolution to infer the stability of MOPS against oxidation by Mn oxide. Although solid-phase Mn reduction was slower by MOPS relative to the other Good’s buffers and MOPS was the only buffer that generated minimal aqueous Mn under all conditions tested, our results demonstrate that up to 30% Mn reduction as solid-phase Mn(III) can occur after 24 h reaction with MOPS buffer (Figs. 1, 2). Recent studies also suggest that PIPES buffer provides a less reactive alternative to HEPES because the N linked to the alkyl sulfonic groups is less reactive than the N linked to the hydroxyl branch^70,71^. While solid-phase Mn(III) generation observed after 1 h reaction with PIPES was less than half that observed in the HEPES treatment under similar pH conditions, the difference in Mn reduction between treatments diminished after 24 h with up to 31 (± 1)% reduction by PIPES and 35 (± 5)% reduction by HEPES (Figs. 1, 2, Supplementary Table S2). However, at higher pH conditions of *ca.* 7.7, significantly less Mn was reduced by PIPES than HEPES with up to 22 (± 3) and 33 (± 1)% total Mn reduction, respectively.

The mechanism of reduction by Good’s buffers likely involves a one electron transfer from the organic molecule to Mn(IV) to form Mn(III) and a radical intermediate^18,19,72^. This mechanism is consistent with our observations, where solid-phase Mn(III) is the dominant reduction product formed. Radical intermediates have been shown to undergo subsequent *N*-dealkylation or C-hydroxylation and quantification of the possible reaction products in future studies may provide additional insight into the reaction mechanism and total number of electrons transferred^73^. The formation of a HEPES radical^18^ is thermodynamically favorable since the HEPES radical/HEPES couple (+ 0.8 V vs. standard hydrogen electrode)^18^ lies below the standard redox potential of Mn^IV^O_2 (s)_/Mn^2+^_(aq)_ of 1.23 V^1,74^. Based on a study of compounds with a piperazine ring^73^, the reaction between HEPES and δ-MnO_2_ likely occurs at the piperazinyl N atom following adsorption of the HEPES molecule. Although MES itself has been reported to not form radical species^17,72^, studies of related organic compounds show radicalization of the morpholine ring^73^. Thus, like HEPES and PIPES, MES and MOPS most likely form a radical intermediate, which renders them ineligible for use in environmental studies involving redox-sensitive species.

The free radical chain reaction mechanism^75^ implicated in Mn reduction by Good’s buffers cannot explain Mn(IV,III) reduction by TRIS buffer since it lacks the ring structure that stabilizes the radical intermediate. Instead, the extensive reduction of Mn by TRIS can be explained by its ability to form complexes with Mn^76^ and the increased reactivity of aliphatic amines relative to ring-bound N^77,78^. Furthermore, complexation of TRIS with Mn(II) may also contribute to the sustained aqueous Mn(II) concentrations^17,79^, while surface complexation of TRIS by δ-MnO_2_ may enhance Mn reduction and facilitate either appreciable second electron transfer and thereby generation of Mn(II) or increased Mn(III) production that favors disproportionation to Mn(II) and Mn(IV).

### Kinetics of Mn(III) generation within δ-MnO_2_ as a function of increasing buffer concentration

**Figure 3:**
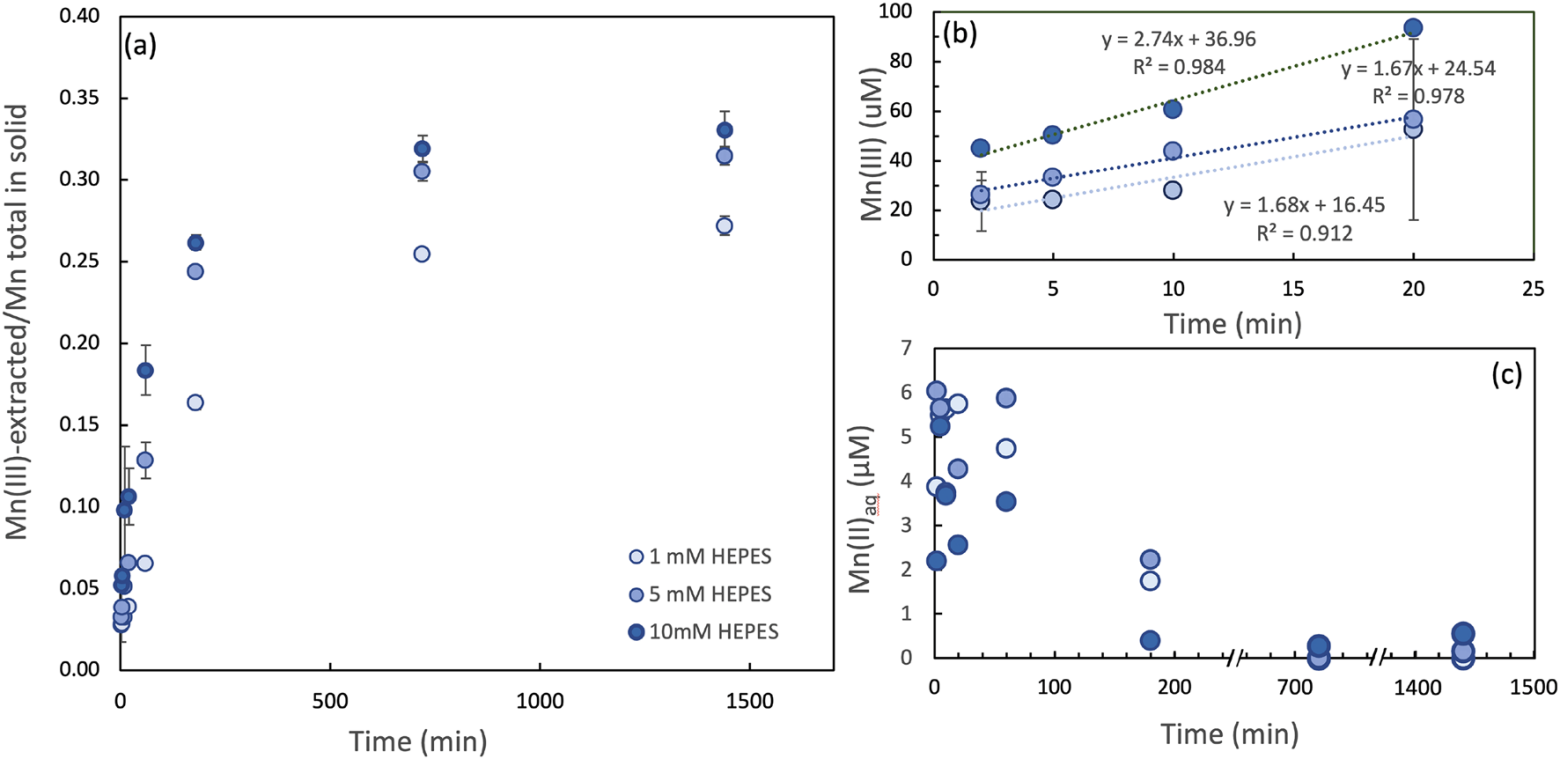
(**a**) Effect of HEPES concentration on the kinetics of Mn reduction in δ-MnO_2_ at pH 7.5 as quantified by pyrophosphate extractions. (**b**) Initial rates of Mn(III) production as a function of total HEPES concentration. (**c**) Aqueous Mn(II) concentration over time; note the axis-break in (**c**). Error bars represent standard deviation of triplicates; when not visible, error bars are smaller than the marker size.

To investigate the effect of Mn:buffer ratio, δ-MnO_2_ was reacted with 1, 5, and 10 mM HEPES buffer at pH 7.5 The initial rate and extent of reduction of Mn(IV) to Mn(III) scaled with the buffer concentration as shown in Fig. 3a,b. As the reaction reached steady state, after *ca.* 24 h, the proportion of solid phase Mn(III) in δ-MnO_2_ was 27.2, 31.5, and 33.1% (mol Mn(III) mol^−1^ Mn) for 1 mM, 5 mM and 10 mM HEPES, respectively (Fig. 3a, Supplementary Table S3). The time required to get to 50% of the steady-state Mn(III) concentration decreased from 148, 87, and 51 min for HEPES concentrations of 1, 5, and 10 mM, respectively (Fig. 3a). The initial rate of Mn(III)-generation ranged from 1.7 to 2.7 μM min^−1^, with the highest reduction rate occurring for the highest buffer concentration (Fig. 3b). In addition, a small spike in aqueous Mn (2–6 µM, < 1% Mn_TOT_) was detected within the first hour of the reaction suggesting that Mn(II), which can originate either from Mn(III) disproportionation or HEPES reduction of Mn(III,IV), becomes adsorbed over time at pH 7.5 (Fig. 3c).

### Susceptibility of different abiotic Mn oxides to manganese reduction

To determine the effect of the initial Mn(III)-content and AMON value on the susceptibility of Mn reduction by HEPES buffer, three different Mn oxides (i.e., δ-MnO_2_, δ-MnO_2_** and c-dis Bi; Table 1) were reacted with 10 mM HEPES at pH 7.5 while maintaining a 10:1 HEPES:Mn_TOT_ molar ratio. After 1 h and 24 h of reaction, Mn(III) accumulation in pure Mn(IV) valence δ-MnO_2_ increased to *ca.* 18 and 33%, respectively. For δ-MnO_2_**, which initially contained 15.2 ± 0.3% Mn(III), the pyrophosphate-extractable Mn(III) content increased about 3 and 8% (for a total of 18 and 23% Mn(III)) after 1 and 24 h, respectively (Supplementary Table S3). In contrast, there was no appreciable increase in the quantity of pyrophosphate-extractable Mn(III) for c-disordered H^+^ birnessite (c-dis Bi) after 1 h of reaction with HEPES (Table 1, Supplementary Table S3).

While the initial amount of Mn(III) influences the reducibility of the mineral, the buffer concentration also matters as shown in Fig. 3. Another photo-reduced δ-MnO_2_, δ-MnO_2_*, which initially contained 13.4 ± 1.4% pyrophosphate-extractable Mn(III), was reacted with 10 mM HEPES at the same pH value but at a lower Mn concentration, which resulted in a 20:1 HEPES: Mn_TOT_ molar ratio. For this sample, the total pyrophosphate-extractable manganese(III) increased to *ca.* 26 and 35% after 1 and 24 h, respectively. These results show that the extent of Mn reduction increases with increasing buffer: Mn_TOT_ molar ratios. Therefore, in addition to pH, both the initial mineral redox state and the buffer : Mn_TOT_ molar ratios must be considered in determining the extent to which the buffer changes mineral composition and redox state.

**Figure 4:**
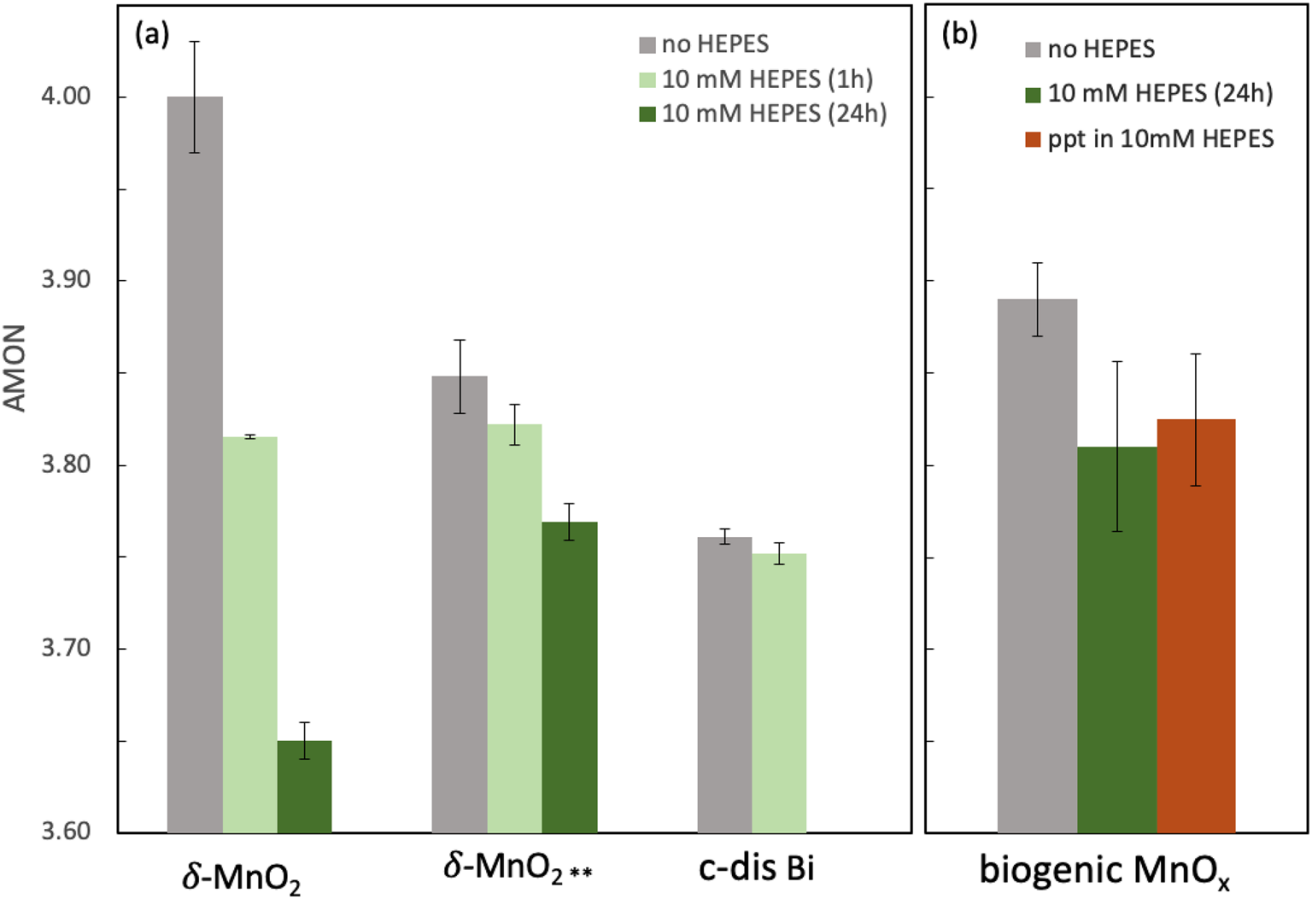
(**a**) Average manganese oxidation number (AMON) for δ-MnO_2_, δ-MnO_2_** [15% Mn(III)] and c-dis Bi [16% Mn(III), 4% Mn(II)] before and after reaction with 10 mM HEPES at pH 7.5 and a 10:1 HEPES:Mn ratio. For δ-MnO_2_**, AMON values are estimated from PP-Mn(III) measurements. (**b**) AMON values for biogenic Mn oxides precipitated in the presence and absence of HEPES at pH 6.8 with a 40:1 HEPES:Mn ratio. Biogenic Mn oxides precipitated in the absence of HEPES (grey bar) were subsequently reacted with 10 mM HEPES (t = 24 ± 4 h) (green bar) and compared with biogenic oxides precipitated in the presence of 10 mM HEPES (brown bar). Error bars represent standard deviation of triplicates.

Figure 4a shows AMON values of the three abiotic Mn oxides, δ-MnO_2_, δ-MnO_2_** and c-dis Bi, before and after 1 and 24 h reaction with HEPES. This allows us to compare these abiotic oxides to biogenic oxides (next section, Fig. 4b) since biogenic oxides are not amenable to pyrophosphate extraction due to complex interactions of pyrophosphate^80^ with the bacterial biomass associated with the oxide particles. For δ-MnO_2_, the initial AMON of 4.0 decreased significantly after both 1 and 24 h, first to 3.82 and then ultimately 3.65. Less reduction was observed for δ-MnO_2_**, where the initial AMON of 3.85 decreased to 3.822, the same AMON as δ-MnO_2_ after 1 h, and then to just 3.77 after 24 h. No significant change in the AMON of c-dissordered birnessite was observed, however, as it remained at *ca.* 3.76 both before and after reaction with HEPES. While δ-MnO_2_** and c-dis Bi contained the same initial amount of Mn(III), the lower reducibility of c-dis Bi may result from the presence of ~ 4% Mn(II) in the solid phase (Table 1) or a difference in the crystallographic distribution of Mn(III) between layer and interlayer positions, which may vary with the mechanism of Mn(III) generation during mineral synthesis or preparation (see “Methods” section).

### Reduction of biogenic Mn oxides

Biogenic manganese oxides typically are nanoscale, layer-type manganese oxides with hexagonal sheet symmetry, abundant octahedral vacancy sites and an average manganese oxidation number of 3.7 to 3.9^20,48,50,52,53^. The biogenic manganese oxide produced by the model Mn oxidizing bacterium, *Pseudomonas putida* (*P. putida*) GB-1, enzymatically oxidizes Mn during the stationary growth phase, such that manganese oxide particles are precipitated extracellularly and enmeshed in a biofilm matrix^20,50,81^. To assess whether abiotic and biogenic oxides are similarly susceptible to reduction by organic buffers, biogenic Mn oxides were exposed to 10 mM HEPES buffer either after or during precipitation. The initial AMON of biooxides formed with 250 µM Mn and without any HEPES buffer was 3.89 ± 0.02, which indicates 89 or 95% Mn(IV) depending on whether we assume that the oxide consists exclusively of Mn(IV) and Mn(III) or Mn(IV) and Mn(II), respectively. After 24 h of reaction with HEPES, the AMON decreased to 3.81 ± 0.05 (Fig. 4b, 40:1 HEPES:Mn ratio). The AMON value of biooxides formed in the presence of HEPES (40:1 HEPES:Mn ratio) was 3.83 ± 0.04, which was similar to the value of the HEPES-reacted biooxides. Even after an additional 10 mM HEPES buffer was added to biooxides formed in HEPES, no change in AMON was observed (Supplementary Table S3). Despite pushing the system toward Mn reduction with a high buffer:Mn ratio of 40, we only observed a moderate decrease in AMON values from 3.9 to 3.8. The lower extent of Mn reduction observed for the biogenic manganese oxides may be due to physical or chemical interactions of the manganese oxides with the surrounding bacterial biomass, which is comprised by *P. putida* GB-1 cells and extracellular polymeric substances^81^, that may limit electron transfer from HEPES to Mn(IV,III). Alternatively, the limited decrease in the AMON value compared to the abiotic Mn oxides could be explained by any bacterial re-oxidation of Mn(II, III) that is generated upon reduction of Mn(IV,III) by HEPES. Due to the difficulty of separating the bacterial biomass from the mineral particles or inhibiting microbial oxidation without impacting the oxidation state of Mn in the oxides, we could not determine the mechanism responsible for the lower extent of Mn reduction compared to that predicted from abiotic analogs.

### Relationship between the initial AMON and extent of Mn(IV) reduction

**Figure 5:**
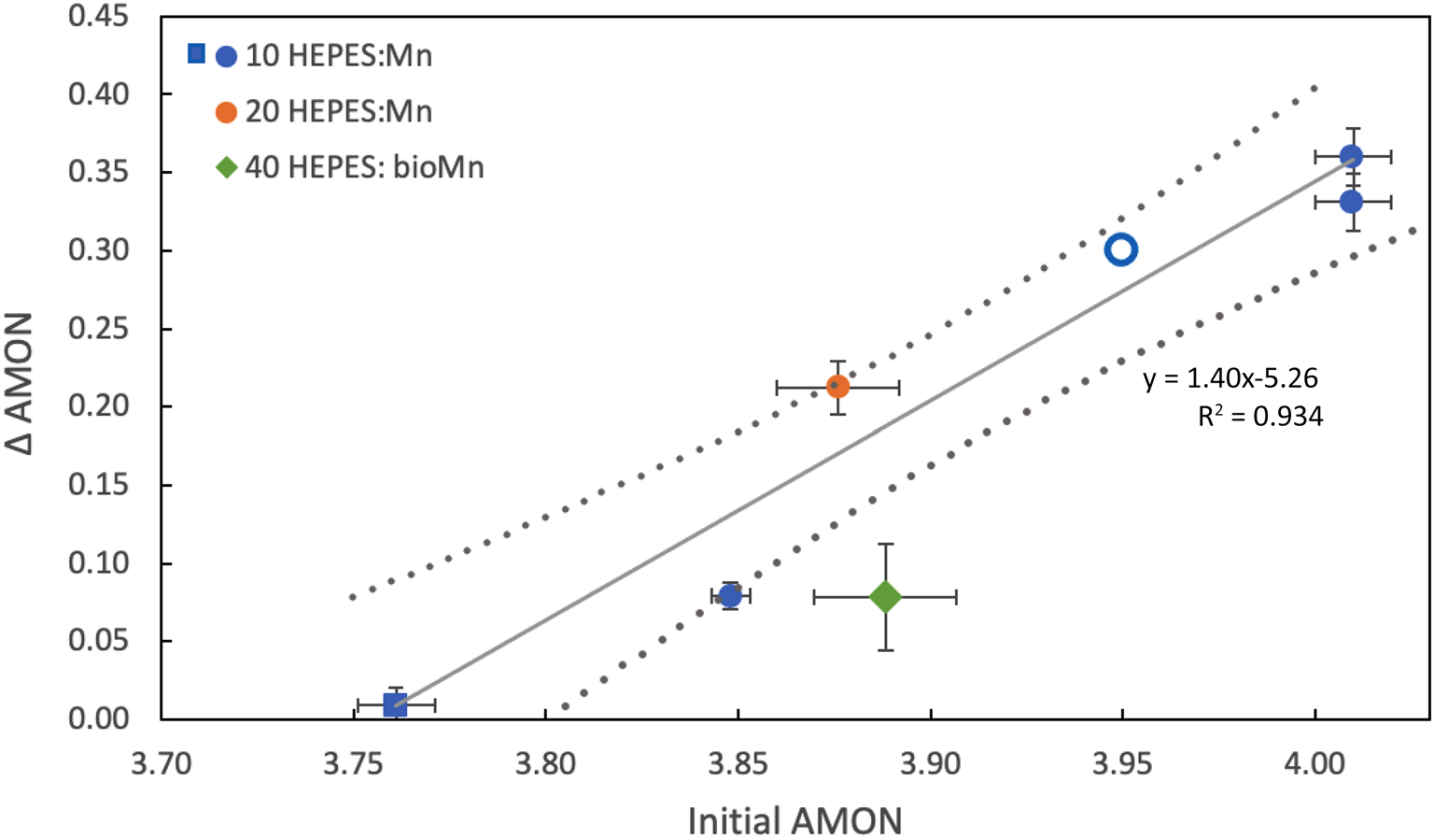
Change in average manganese oxidation number (AMON) as a function of the initial AMON value for abiotic and biogenic Mn oxides. δ-MnO_2_ (circles) and c-dis Bi (square) were reacted with HEPES (pH 7.5), while biogenic Mn oxides were reacted at pH 6.8. Open marker represents literature values from Simanova et al.^58^; see Supplementary Table S5 for a summary of sample information. Horizontal error bars represent the standard deviation between triplicate samples (except for c-disordered birnessite, which was run in duplicate) with vertical error bars calculated following the simple rule for sums and differences and the overlay (grey dotted line) shows the 95% confidence interval.

In Fig. 5, we synthesize our data to show the change in AMON value as a function of initial AMON value for abiotic and biogenic oxides reacted with an excess of HEPES buffer (> 10 HEPES: Mn_TOT_ molar ratio, pH 7.5) together with available literature values (Supplementary Table S5). Overall, this data compilation shows that the initial AMON value is a strong indicator of the susceptibility of the mineral to reduction: minerals with lower AMON values are less susceptible to reduction by organic buffers. Manganese reduction in biogenic Mn oxides was lower than predicted from the abiotic trendline notwithstanding the high HEPES:Mn ratio in biogenic MnO_2_ relative to abiotic Mn oxides and the presence of a biofilm matrix rich in reduced carbon moeities. The hypothesis proposed in the previous section—that bacterial re-oxidation of Mn(II)/Mn(III) generated through HEPES reduction may explain the muted decrease in AMON values of biogenic relative to abiotic oxides is also supported by the low position of the biogenic Mn oxides in Fig. 5. Accordingly, our results suggest that the presence of an active Mn oxidizing culture plays a critical role in maintaining the redox state of biogenic Mn oxides.

### Implications

Knowledge on the formation, structure, and reactivity of Mn oxides is generally derived from studies of model systems that use chemical buffers for pH control. This work showed that organic buffers, including morpholinic and piperazinic Good’s buffers, will bias results towards lower reactivity and reducibility of Mn oxides due to extensive decrease in AMON and increase in solid-phase Mn(III). The extent of Mn reduction in biogenic Mn oxides following prolonged interaction with HEPES buffer was lower than that predicted from abiotic experiments suggesting that organo-mineral interactions and/or continued biogenic activity play a critical role in the reactivity of these biominerals.

Studies that continue to employ Good’s buffers must ensure characterization of Mn oxides and run paired controls without relying on the accumulation of aqueous manganese to detect Mn reduction. Given that protocols for wet-chemical measurements of solid-phase Mn(III) content and average manganese oxidation number are available, as used in this study, we encourage that these measurements be included in studies of abiotic or biogenic manganese oxides in order to account for changes in reactivity associated to changes in Mn(III) content and/or AMON. Additionally, due to the range of factors that influence the reactivity of Mn oxides (i.e., Mn(III)-content and mineral structure, buffer:Mn_TOT_ ratio, pH, presence of microbial biomass), Mn reduction by organic buffers cannot be predicted by a single variable and alternative options for pH control are strongly recommended. Whenever possible, we recommend that studies investigating redox-active minerals use a pH stat for pH control and avoid the use of organic buffers. In order to maximize throughput, experiments can be transitioned to manual pH monitoring and control after initial timepoints. Depending on the study type, inorganic buffers may be less problematic than organic buffers. Additionally, for inorganic buffers, ion sorption can be readily measured in order to determine its potential impact on surface reactivity. Working without buffers will be more challenging for generating biogenic manganese oxides since pH control is important in the propagation of microbial cultures. The use of buffers with manganese oxidizing bacteria may be less likely to bias results given their lower AMON values and reducibility; however, appropriate measures should be taken in order to account for buffer effects. In fungal systems, Good’s buffers not only influence the composition of the mycogenic manganese oxides, but appear to interfere with enzymatic manganese oxidation^82^. The interaction between organic buffers and Mn oxides also provides insight into the potential for naturally-occurring organic molecules with similar functional groups (i.e., sulfonic acids)^82^ to lower the redox state of manganese oxides. Finally, this study further challenges the assumption that the absence of aqueous Mn production indicates the absence of Mn reduction and underscores the need to quantify solid-phase Mn reduction in addition to Mn reduction that results in the release of Mn to solution. This approach will provide an improved understanding of the role played by Mn oxides in driving important biogeochemical processes implicated in carbon, nutrient and contaminant cycling.

## Methods

All solutions were prepared using ultrapure (18 MΩ-cm) water and A.C.S. reagent-grade chemicals.

### Mineral preparation

#### Abiotic MnO_2_ synthesis

δ-MnO_2_ was synthesized by reacting solutions of Mn^VII^ (KMnO_4_) and Mn^II^ (MnCl_2_) at a ratio of 0.67 under alkaline conditions according to Marafatto et al. The suspension was washed in NaCl to exchange K^+^ for Na^+^ as the interlayer cation before finally being washed in MQ water to remove any excess Na^+^. The same protocol was used to produce c-disordered H^+^ birnessite (c-dis Bi) but with a Mn^VII^/Mn^II^ ratio of 0.52^7,83^. After synthesis, stock suspensions were stored at 20 °C. To prepare δ-MnO_2_ enriched in Mn(III) without the use of any chemical reductants_,_ a suspension of δ-MnO_2_ (10 mM NaCl, 0.3 mM Mn at pH 7.2) was recirculated through a quartz cuvette and irradiated for 10 days using an array of 1-W light-emitting diodes at 400 nm (3.1 eV)^84^. The AMON of initial oxide suspension (δ-MnO_2_) was measured via potentiometric titration, whereas solid-phase Mn(III) was quantified by a sodium pyrophosphate (Na-PP) extraction and UV–Visible spectrophotometry (Table 1). The properties of these oxides, including AMON and solid-phase Mn(III) content, are provided in Table 1.

#### Biogenic MnO_2_ synthesis

Biogenic Mn oxides were produced using *Pseudomonas putida* (*P. putida*) GB-1 biomass (0.4–0.6 g_dry mass_ L^−1^)^50,56,85^ in the absence or presence of 10 mM HEPES with pH maintained at 6.8 ± 0.2 (Metrohm 718 Titrino or 906 Titrando)^29^. All microbiological work was conducted in a sterile laminar flow-hood. Growth medium (*Leptothrix* medium) was prepared by dissolving medium components in MQ water, autoclaving (20 min, 120 °C), and adding filter-sterilized metal cation solutions once the autoclaved solution cooled to room temperature. *Leptothrix* medium is a nutrient-rich growth medium containing 1.0 g L^−1^ d-glucose, 0.5 g L^−1^ yeast extracts, 0.5 g L^−1^ casamino acids, 2.38 g L^−1^ HEPES acid, 0.5 mM CaCl_2_, 0.83 mM MgSO_4_, 3.7 μM FeCl_3_, 250 μM MnCl_2_, 40 nM CuSO_4_·5H_2_O, 152 nM ZnSO_4_·7H_2_O, 84 nM CoCl_2_·6 H_2_O and 54 nM Na_2_MoO_4_·2H_2_O^21﻿,86^.

An overnight culture was prepared from a frozen *P. putida* GB-1 stock culture (− 80 °C), which was transferred to *Leptothrix* medium without Mn and incubated for 13 h at 27 °C, 150 RMP until an OD_600_ ~ 0.6 A.U. (as measured by portable UV-spec). Then 130 μL of *P. putida* was inoculated into 250 mL Erlenmeyer flasks containing 130 mL medium. After 20 h (OD = 0.9), the biomass was rinsed 3 times with 10 mM NaCl (4000×*g*, 150 mm rotor). The supernatant after initial centrifugation was reserved for use in later experiments (hereafter referred to as spent growth medium). The biomass was then either re-suspended in an electrolyte solution (0.5 mM CaCl_2_, 0.83 mM MgSO_4_) or spent growth medium and 250 μM Mn. To precipitate the oxides, the biomass from several flasks was pooled (600 mL) and transferred to a 1 L flask. The flask contents were stirred continuously in a water bath at 27 °C; pH was kept constant (6.8 ± 0.2) using a Metrohm 718 Titrino or 906 Titrando and/or 50 mM NaOH and 50 mM HCl). After 48 h, biogenic MnO_2_ was either characterized according to the AMON value or used in further experiments as described below.

### Abiotic and biogenic batch experiments

#### Mn reduction by common buffers

To determine the importance of buffer structure on Mn reduction, δ-MnO_2_ suspensions (~ 1 mM Mn) were reacted with 10 mM MES, PIPES, MOPS, HEPES, and TRIS buffer. These buffers have pKa values equal to 6.10, 6.76, 7.28, 7.48, and 8.06 respectively. Experiments were performed at pH values equal to the pK_a_, pK_a_ + 1, and pK_a_ − 1 for each buffer. In general, sample aliquots were collected after 1 h and 24 h of reaction. Solid-phase Mn(III) was quantified by sodium-pyrophosphate extractions, and inductively coupled plasma optical emission spectrometry (ICP-OES) was used to quantify concentrations of aqueous (assumed to be synonymous with Mn(II) for this study) and solid-phase Mn as described below. Due to reductive dissolution of synthetic Mn oxides reacted with TRIS buffer during pyrophosphate extraction of Mn(III), likely promoted by TRIS adsorption to the oxide, potentiometric titrations were used to determine AMON and estimate the Mn(III) content of TRIS-reacted δ-MnO_2._

#### Kinetics of Mn reduction by HEPES

In order to measure the rate of Mn(IV,III) reduction by HEPES, additional experiments were run at pH 7.5 (± 0.1) using ~ 1 mM δ-MnO_2_ with 1, 5, and/or 10 mM HEPES and 10 mM NaCl. The solid phase was sampled, washed and analyzed for pyrophosphate extractable Mn(III) for a 24 h time-course at 0, 5, 10, 20, 60, 180, 720, and 1440 min^7^. Samples were also collected to determine both total and aqueous Mn concentrations using ICP-OES.

#### Influence of initial AMON and presence of biomass on Mn reduction by HEPES

To assess the effect of initial Mn valence state on the extent of Mn reduction, δ-MnO_2_ containing 13 and 15.2% Mn(III) (referred to as δ-MnO_2_* and δ-MnO_2_**, respectively) and c-dis Bi containing 15.6% Mn(III) and 4% Mn(II) were reacted with 10 mM HEPES at pH 7.5 (Supplementary Table S3). Total and aqueous Mn concentrations as well as Mn(III)-generation was measured after 1 and 24 h as described above. Finally, biogenic Mn oxides were precipitated in the presence and absence of HEPES (10 or 0 mM HEPES, 0.25 mM Mn) for 48 h. Biogenic Mn oxides precipitated in the absence of HEPES were then reacted with 10 mM HEPES at pH 6.8 for 24 (± 4) h. Mn(III) generation in bio-oxides was not quantified with the pyrophosphate-extraction method since it is not yet fully developed for use with biogenic Mn oxides, but the AMON was determined at the end of the reaction period.

### Mn characterization

Total and aqueous manganese concentrations were measured in triplicate by inductively coupled plasma optical emission spectrometry (ICP-OES, Perkin–Elmer Optima 8300) at three different emission wavelengths (259.372, 257.610 and 260.568). Eight standard solutions ranging from 0.5 to 500 μM were prepared from 1000 mg/L Perkin–Elmer single element standards. Measured intensities were normalized relative to a 50 ppm Sc internal standard. For total Mn analysis, one milliliter of the Mn suspension was digested in 9 mL of 3% HNO_3_ and 0.1 M oxalic acid.

### Redox state determination

Solid-phase Mn(III) content was quantified for all abiotic samples except those reacted with TRIS buffer. Average Mn oxidation numbers (AMON) were determined by potentiometric titration for both abiotic and biotic samples, unless otherwise noted. For samples analyzed by both pyrophosphate extractions (i.e., solid-phase Mn(III) content) and potentiometric titration (AMON), solid phase Mn(II) was calculated by difference, since AMON = 4x + 3y + 2z, where x + y + z = 1 and y is determined directly from pyrophosphate extraction. For samples where z = 0 and y was determined by pyrophosphate extraction, AMON can be estimated from AMON = 4x + 3y.

#### Pyrophosphate extraction

We used sodium pyrophosphate to extract Mn(III) from Mn oxides^87^. To initiate the extraction, 8 mL of slurry was collected on a 0.22 μm filter membrane (Filtropur S, Sarstedt) and rinsed three times with 10 mM NaCl. The filter was submerged in 8 mL MQ water and sonicated for 5 min to re-suspend the particles. The filter was then removed with tweezers and 2 mL of 120 mM Na-pyrophosphate (pH 6.5) was added. Test tubes were covered with aluminum foil and placed on an end-over-end shaker. After 48 h, a 1 mL aliquot was taken for determination of total Mn concentration. An additional 4 mL were filtered through 0.2 μm nylon filters and the Mn(III)-pyrophosphate concentration in the filtrate determined by UV–Vis spectrophotometry at 258 nm. Total and aqueous Mn concentrations of all pyrophosphate extractions were measured using ICP-OES as described above. This method could not be used for TRIS-reacted δ-MnO_2_ or biogenic Mn-oxides precipitated in the presence of bacterial biomass within 2 days of reaching stationary phase because the addition of pyrophosphate stimulated reductive dissolution of the oxides.

#### Potentiometric titration

Average Mn oxidation number (AMON) was determined by a three-step titration^88,89^. This method yields a concentration independent measure of the average Mn oxidation state^58^. Samples for AMON determination were obtained by collecting the solids from 90 mL of slurry onto a filter membrane by vacuum filtration. The solids were rinsed three times using 10 mM NaCl and subsequently dissolved in 40 mL of 0.02 M Mohr’s salt ((NH_4_)_2_Fe(SO_4_)_2_·6H_2_O) solution. The same titration was performed on biogenic Mn oxides although additional sample preparation was required to remove any interference from associated organic compounds originating from the bacterial biomass. Specifically, the solids collected from about 600 mL of the biogenic manganese oxide suspension were rinsed three times with 10 mM NaCl through cyclic centrifugation and re-suspension before being directly dissolved in 50 mL of 0.02 M Mohr’s salt solution. After oxide dissolution, the slurry was passed through two 0.2 μM filtroporus filters (Sarstedt) and a Dionex On guard™ II RP filter to remove organic detritus. All of the Mohrs salt was recovered by rinsing implicated glassware and filters three times with 15 mL of 10 mM NaCl. The filtrate was then titrated as described below.

The titration was done using a Metrohm 888 Titrando automatic titrator equipped with a Pt potentiometric electrode. First, a reference solution of Mohr’s salt within 0.004 g was titrated with KMnO_4_ in order to determine the total concentration of Fe^2+^ ions. Next, the solution containing the dissolved Mn oxide (and the same number of moles of Fe^2+^ as the reference solution) was titrated with KMnO_4_ in order to quantify the amount of Fe^2+^ oxidized during Mn reduction by Mohr’s salt. Sodium pyrophosphate (Na_4_P_2_O_7_) was then added to the titrated solution in excess and pH was adjusted to 6.5 with 6N NaOH. A final titration with KMnO_4_ was then used to determine the total amount of Mn^2+^ (both present within the oxide and formed during the first titration). As this method of titration is based on the measurement of these three equivalence volumes, it is not reliant on sample mass or the concentration of titrating solution and reproducibility error stems only from the difference in volumes of Mohr salt between the reference and sample solution^89^.

## Supplementary Information

Supplementary Tables.

## Data Availability

All data generated or analyzed during this study are included in this published article (and its Supplementary Information file) and at 10.5281/zenodo.7834812.
