# Supplementary material for: Organic buffers act as reductants of abiotic and biogenic manganese oxides
Source: Sci Rep. 2023 Apr 20;13:6498. doi: 10.1038/s41598-023-32691-5 (PMC10119380; doi:10.1038/s41598-023-32691-5)
Supplement: Supplementary file 1 — Supplementary Tables. [file 41598_2023_32691_MOESM1_ESM.pdf]

Scientific Reports Article

Organic buffers act as reductants of abiotic and biogenic manganese oxides

Debra M. Hausladen[1,2] and Jasquelin Peña[1,3*]

1. Institute of Earth Surface Dynamics, University of Lausanne, 1015 Lausanne, Switzerland
2. Department of Civil and Building Engineering, Université de Sherbrooke, QC J1K 2R1, Canada
3. Department of Civil and Environmental Engineering, University of California, Davis, CA 95616

*corresponding author: pena@ucdavis.edu

Supplementary Information

Number of Pages: 6
Number of Tables: 5

Table S1. Summary of Good's and TRIS buffers used in this study

Buffer	Type	Family	pK_a	Structure
MES	Good's buffers	Morpholinic	6.10	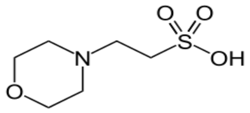
MOPS			7.28	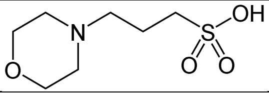
PIPES		Piperazinic	6.76	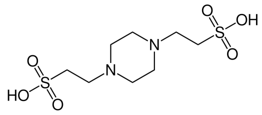
HEPES			7.48	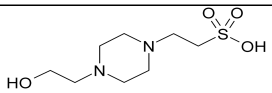
TRIS	TRIS	TRIS	8.06	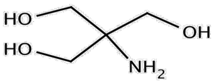

Table S2. Summary of experiments where δ-MnO_2 was reacted with MOPS, MES, PIPES, HEPES, and TRIS buffers (Figures 1 and 2), including extent of Mn reduction as quantified by potentiometric titration (AMON) and/or pyrophosphate extraction (Mn(III)-PP). $Mn(II)_{aq}$ represents the aqueous Mn concentration of filtered suspensions. Uncertainty reflects standard deviation between triplicates (Mn(III)-PP and $Mn(II)_{aq}$) or duplicates (AMON).

Abiotic	pH	Mn_{total} (mM)	Buffer	Time	AMON	Mn(III)-PP (%)	$Mn(II)_{aq}$ (μM)
δ-MnO_2	6.24	0.41 ± 0.03	10 mM MOPS	1 h		15 ± 1	2.2 ± 0.2
	6.25	0.41 ± 0.01	10 mM MOPS	24 h		30 ± 1	0.23 ± 0.01
	7.25	0.424 ± 0.003	10 mM MOPS	1 h		13.4 ± 0.1	1.8 ± 1.5
	7.25	0.41 ± 0.01	10 mM MOPS	24 h		29 ± 1	0.1 ± 0.1
	8.18	0.429 ± 0.004	10 mM MOPS	1 h		9.7 ± 0.3	4.1 ± 0.4
	8.12	0.41 ± 0.01	10 mM MOPS	24 h		17 ± 4	0.75 ± 0.06
	5.1	0.42 ± 0.02	10 mM MES	1 h		29.8 ± 1	0.35 ± 0.06
	5.1	0.503 ± 0.108	10 mM MES	24 h		33 ± 3	23 ± 1
	6.1	0.747 ± 0.163	10 mM MES	1 h		22.6 ± 2	0.14 ± 0.01
	6.1	0.612 ± 0.94	10 mM MES	24 h		23.8 ± 2	1.6 ± 0.1
	7.0	0.788 ± 0.168	10 mM MES	1 h		17.0 ± 1	0.4 ± 0.1
	7.0	0.663 ± 0.150	10 mM MES	24 h		24.0 ± 1	1 ± 1
	5.84	0.955 ± 0.032	10 mM PIPES	1 h		28.4 ± 0.2	0.80 ± 0.05
	5.84	1.030 ± 0.020	10 mM PIPES	24 h		33.8 ± 1.8	49 ± 1
	6.82	0.997 ± 0.022	10 mM PIPES	1 h		14.6 ± 1.3	0.37 ± 0.08
	6.82	1.054 ± 0.017	10 mM PIPES	24 h		31.0 ± 0.8	0.32 ± 0.03
	7.73	1.058 ± 0.008	10 mM PIPES	1 h		6.0 ± 0.3	0.43 ± 0.17
	7.72	1.022 ± 0.039	10 mM PIPES	24 h		21.8 ± 3.3	0.034 ± 0.001
	6.7	0.720 ± 0.148	10 mM HEPES	1 h		34 ± 6	0.84 ± 0.06
	6.7	0.853	10 mM HEPES	24 h		35 ± 5	21 ± 1
	7.6	0.912 ± 0.02	10 mM HEPES	1 h	3.82 ± 0.01	18 ± 2	4 ± 3
	7.6	0.904 ± 0.02	10 mM HEPES	24 h		33 ± 1	1 ± 0
	8.5	0.837 ± 0.77	10 mM HEPES	1 h		10 ± 1	ND
	8.5	0.763 ± 0.85	10 mM HEPES	24 h		21 ± 1	ND
	7.1	1.232 ± 0.016	10 mM TRIS	1 h		NA	6 ± 1
	7.1	1.232 ± 0.016	10 mM TRIS	24 h	3.60 ± 0.03	NA	185 ± 3
	8.1	1.209 ± 0.015	10 mM TRIS	1 h		NA	21 ± 2
	8.1	1.209 ± 0.015	10 mM TRIS	24 h	3.57 ± 0.02	NA	23 ± 2
	9.1	1.211 ± 0.032	10 mM TRIS	1 h		NA	21 ± 2
	9.1	1.211 ± 0.032	10 mM TRIS	24 h	3.63 ± 0.02	NA	1.18 ± 0.04

Table S3. Summary of experiments where abiotic and biogenic Mn oxides were reacted with HEPES buffer (Figures 4 and 5), including extent of Mn reduction as measured by potentiometric titration (AMON) or pyrophosphate extraction (Mn(III)-PP). $Mn(II)_{aq}$ represents the aqueous Mn concentration of filtered suspensions. Uncertainty reflects standard deviation between triplicates (Mn(III)-PP and $Mn(II)_{aq}$) and duplicates (AMON). (ND, not detected.)

Abiotic	pH	Mn_{total} (mM)	HEPES	Time	AMON	Mn(III)-PP (%)	$Mn(II)_{aq}$ (μM)
cdBi			0 mM		3.76 ± 0.01	15.6 ± 0.1	2.0 ± 0.2
	7.5	0.967	10 mM	1h	3.75 ± 0.01	15.2 ± 0.9	0.24 ± 0.02
δ-MnO_2**			0 mM			15.2 ± 0.3	0.16 ± 0.01
	7.5	0.963	10 mM	1h		17.8 ± 1.1	0.15 ± 0.01
	7.5	0.963	10 mM	24h		23.1 ± 0.5	0.34 ± 0.04
δ-MnO_2*			0 mM			13.4 ± 1.4	0.45 ± 0.06
	7.5	0.487	10 mM	1 h		25.9 ± 0.1	0.13 ± 0.01
	7.5	0.487	10 mM	24h		34.6 ± 1.0	0.13 ± 0.02
	6.8	0.493	10 mM	1 h		34.8 ± 1.1	0.16 ± 0.03
	6.8	0.493	10 mM	24 h		35.3 ± 4.8	7 ± 3
δ-MnO_2			0 mM		4.01 ± 0.01	3 ± 1.4	0.5 ± 0.4
	7.5	1.205	1 mM	1 h	3.92 ± 0.01	8.7 ± 0.3	10 ± 1
	7.5	0.861	1 mM	1 h		6.6 ± 0.3	5 ± 4
	7.5	0.913	5 mM	1 h		12.9 ± 1.1	6 ± 1
	7.5	0.899	10 mM	1 h		18.4 ± 1.5	4 ± 3
	7.5	1.180	10 mM	1 h	3.82 ± 0.01	18.55 ± 0.07	11 ± 2
	7.5	0.861	1 mM	24 h		27.2 ± 0.8	ND
	7.5	0.913	5 mM	24 h		31.5 ± 1.0	0.15 ± 0.01
	7.5	0.899	10 mM	24 h		33.1 ± 1.1	0.56 ± 0.02
Biogenic		$Mn(II)_{initial}$					
	6.8	0.25	0 mM		3.90 ± 0.01		0.1
	6.8	0.25	10 mM	1 h	3.86		
	6.8	0.25	10 mM	28 h	3.86		ND
	6.8	0.25	0 mM		3.87		
	6.8	0.25	10 mM	24 h	3.77		
	6.8	0.25	0 mM		3.90		0.1
	6.8	0.25	10 mM	24 h	3.80		
	6.8	0.25	10 mM (ppt)	48 h	3.83 ± 0.04		ND
	6.6	0.25	+ 10 mM	1 h	3.84		0.4
	6.8	0.98	10 mM (ppt)	5 d	3.72		

Table S4. Total and aqueous Mn concentrations of TRIS-reacted δ-MnO_2 ($AMON_{initial}$ = 4.0; pH = ± 1 pKa) suspensions over time compared to results from pyrophosphate (PP) extraction of sample aliquots collected. Particles used for pyrophosphate extractions were rinsed with copious amounts of water before addition of sodium pyrophosphate. Uncertainty reflects standard deviation between triplicates.

		Pre-PP extraction		PP extractions		
pH	Time (min)	Total Mn (μM)	Mn_{aq} (μM)	Total Mn (μM)	Mn_{aq} (μM)	Mn(III) (μM)
7.07	**1**	1232 ± 16	14 ± 0.5	873 ± 249	776 ± 299	803 ± 321
	**10**		18 ± 4	929 ± 313	522 ± 515	827 ± 267
	**60**		6 ± 1	1221 ± 12	1083 ± 59	942 ± 9
	**1440**		185 ± 3	1073 ± 9	906 ± 12	776 ± 21
8.07	**1**	1209 ± 15	18 ± 4	1106 ± 38	1044 ± 22	986 ± 22
	**10**		21 ± 1	1110 ± 55	1015 ± 60	965 ± 51
	**60**		21 ± 2	1132 ± 123	1078 ± 141	918 ± 124
	**1440**		23 ± 2	1199 ± 21	1105 ± 10	997 ± 19
9.07	**1**	1211 ± 32	23 ± 2	1182 ± 37	861 ± 47	847 ± 45
	**10**		20 ± 1	1138 ± 83	851 ± 193	806 ± 162
	**60**		21 ± 2	1244 ± 125	1055 ± 184	915 ± 164
	**1440**		1.18 ± 0.04	1185 ± 10	1196 ± 151	999 ± 19

Table S5. Summary of measured and literature[4,5] values of abiotic and biogenic Mn oxides including those plotted in Figure 5. Change in AMON was calculated as the difference between the AMON of abiotically- or biogenically-synthesized Mn oxides suspended in electrolyte solution and the AMON after reaction with organic buffer.

	Mineral	fMn(III) (t = 0)	AMON (t = 0)	sd	fMn(III) (t = 1h)	AMON (t = 1h)[a]	sd	Δ AMON (t = 1h)	sd	fMn(III) (t = 24h)	AMON (t = 24h)[a]	sd	Δ AMON (t = 24h)	sd	pH	Initial [Mn] (mM)	Reductant	buffer:Mn
	δ-MnO_2		**4.01**	**0.01**		3.82	0.01	0.20	0.01	0.350	*3.65*	*0.02*	**0.36**	**0.02**	7.5	1.2	10 mM HEPES	8.3
	δ-MnO_2		4.01	0.01	0.87	*3.13*	*0.01*	0.88	0.01							1.2		
	δ-MnO_2		4.01	0.01	0.066	*3.93*	*0.01*	0.08	0.01	0.272	*3.73*	*0.01*	0.27	0.01	7.5	0.8	1 mM HEPES	1.2
	δ-MnO_2		4.01	0.01	0.129	*3.87*	*0.01*	0.14	0.02	0.315	*3.69*	*0.01*	0.32	0.01	7.5	0.9	5 mM HEPES	5.6
	δ-MnO_2		**4.01**	**0.01**	0.184	*3.82*	*0.01*	0.19	0.01	0.331	*3.67*	*0.01*	**0.33**	**0.01**	7.5	0.9	10 mM HEPES	11.1
Synthetic MnO	δ-MnO_2		4.01	0.01	0.17	*3.83*	*0.01*	0.18	0.01	0.240	*3.76*	*0.01*	0.24	0.01	7	0.7	10 mM MES	13.8
	δ-MnO_2**	0.152	***3.85***	***0.01***	0.178	*3.82*	*0.02*	0.03	0.02	0.231	*3.77*	*0.01*	**0.08**	**0.01**	7.5	1.0	10 mM HEPES	10.4
	δ-MnO_2*	0.134	***3.87***	***0.02***	0.259	*3.74*	*0.01*	0.13	0.02	0.346	*3.65*	*0.01*	**0.21**	**0.02**	7.5	0.5	10 mM HEPES	20.5
	δ-MnO_2*	0.134	*3.87*	*0.02*	0.348	*3.65*	*0.03*	0.21	0.03	0.353	*3.65*	*0.05*	0.22	0.05	6.8	0.5	10 mM HEPES	20.3
	cdBi		**3.761**	**0.01**		3.75	0.01	0.01	0.01		/				7.5	1.0	10 mM HEPES	10.3
	Experiment 1		3.90	0.01		3.86		0.05			3.86		0.04		6.8	0.25	10 mM HEPES	40
Biogenic	Experiment 2		3.87			/					3.77		0.10		6.8	0.25	10 mM HEPES	40
	Experiment 3		3.90	0.01		/					3.80		0.10		6.8	0.25	10 mM HEPES	40
	Average		**3.89**	**0.02**							3.81	0.05	**0.08**	**0.03**				

**Literature values**		Time	fMn(III) (t = 0)[b]	AMON (t = 0)	sd		fMn(III) (t = 24)[b]	Reacted AMON	sd	Δ AMON	sd	pH	[Mn] (mM)	Reductant	buffer:Mn
Hinkle et al., 2016	δ-MnO_2	25 d	0.02	3.98	0.04		0.15	3.69	0.04	0.29	0.06	7.0	16.8	1 mM MES	0.06
Hinkle et al., 2016	cdBi	25 d	0.17	3.79	0.04		0.17	3.72	0.04	0.07	0.06	7.0	20.8	1 mM MES	0.05
Simanova et al., 2015	δ-MnO_2	1 h	0.03	**3.95**	**0.05**		0.07	3.65	0.05	**0.30**	**0.07**	6.6	5.7	10 mM HEPES	1.8

[a] AMON values in italics calculated from solid-phase Mn(III) content determined using PP-extractions; AMON calculations of δ-MnO_2 assume no solid-phase Mn(II) due to agreement between Mn(III)-PP and AMON measurements

[b] Percentage of Mn(III) determined by linear combination fits of XANES spectra
